# Time, Quality, and Integrity: Temporal Autonomy as a Missing Link in Research Assessment Reform

**DOI:** 10.1007/s11948-026-00588-x

**Published:** 2026-02-27

**Authors:** Mads P. Sørensen, Marina Lambert, Tine Ravn, Filip Vostal

**Affiliations:** 1 The Danish Centre for Studies in Research and Research Policy, Department of Political Science, Aarhus University, Aarhus, Denmark; 2 Centre for Science, Technology, and Society Studies (STSS), Institute of Philosophy of The Czech Academy of Sciences, Jilska 1, Prague 1, 110 00 Czech Republic

**Keywords:** Research integrity, Research quality, Acceleration, QRPs, Research assessment, Reform movement

## Abstract

This paper analyses temporality and the experience of increased speed in contemporary academia and its perceived influence on knowledge production. The study is grounded in theories of acceleration-related change in academia and examines the perceived effects of accelerated research processes on contemporary knowledge production, offering insights into the widespread use of questionable research practices (QRPs). Drawing on 36 focus group interviews with 172 researchers across seven European countries, the analysis reveals strikingly consistent perceptions of current reward and merit systems in academia, transcending differences in countries, disciplines, gender, and levels of seniority. These systems are widely perceived to reward speed and quantity at the expense of quality and integrity, thereby potentially undermining the robustness of the knowledge produced. The study thus shows a need for a stronger qualitative component in the assessment system, which aligns with the aims of the ‘research assessment reform movement’ (cf. DORA, CoARA). However, this paper argues that the research assessment reform movement needs to consider temporality in its efforts to reform research assessments. A stronger focus on ‘process time’ and ‘temporal autonomy’ is recommended to ensure that researchers and research units have adequate time to carry out all research tasks while maintaining integrity.

**Supplementary Information:**

The online version contains supplementary material available at 10.1007/s11948-026-00588-x.

## Introduction

Over the last 15 years, a reform movement aimed to change the way research and researchers are assessed has developed (Rushforth & Hammarfelt, 2024). This research assessment reform movement consists of a broad alliance of researchers, institutions, and research reform organisations and initiatives (see e.g. Curry et al., 2020). One of the movement’s defining features is its recommendation to research institutions, funders, and academics to focus less on quantitative metrics and place more emphasis on qualitative peer reviews, when performing research assessments. These recommendations are relayed in several key documents underpinning the movement. The Declaration on Research Assessment (DORA) was created by the American Society for Cell Biology, following a conference in San Francisco in 2012 (DORA, 2024). It stresses the importance of moving away from journal-based metrics, such as the Journal Impact Factor, when assessing the quality of research, and has been signed by more than 26,000 individuals and organizations in 166 countries (DORA, 2025). The Leiden Manifesto, which lists 10 principles for more responsible research assessments, is another important document for the movement (Hicks et al., 2015). A third central document is the Hong Kong principles created at the 6th World Conference on Research Integrity (WCRI) in Hong Kong in 2019 (Moher et al., 2020). The latter especially underlines the importance of research quality in the assessment process.

In general, the research assessment reform movement has been successful in placing the need for reform on the policy agenda. The best example of this is the newly (2022) formed Coalition for Advancing Research Assessment (CoARA), which is connected to the European Research Area (ERA), i.e. the European Union’s (EU) attempt to create a single, borderless market for research, innovation, and technology. As of April 2025, 832 research performing organisations and funders have joined CoARA by signing the Agreement on Reforming Research Assessment (CoARA, 2025). They commit to actively and constructively reform research assessment practices, which includes recognising that research assessments must primarily be based on “qualitative judgement, for which peer review is central” and only should be supported by quantitative indicators when this can be done in a responsible way (CoARA, 2024 p.2). Another fundamental principle in the Agreement is to “Comply with ethics and integrity rules and practices, and ensure that ethics and integrity are the highest priority, never compromised by any counter-incentives” (CoARA, 2024, p.3).

While the research assessment reform movement has foregrounded the need for a fundamental revision of existing research assessment practices and proposed new principles for reshaping the policy agenda – emphasising the need for a stronger focus on research quality assessed through qualitative assessment procedures instead of quantitative metrics – it has largely overlooked the role of temporality in research and its impact on research quality and integrity.

The Hong Kong Principles briefly address the need to accommodate diverse types of research and allowing for different timeframes of assessment. It warns that a lack of diversity could slow down research progress: “Incentives that encourage one fixed idea of the ‘right kind’ of research will slow down, or even stall, progress.” (Moher et al., 2020). The document further recognizes the pitfall of the short-term nature of academic reward cycles in skewing the institutional and funding landscapes to systematically disadvantage research that “builds on chance findings or curiosity-driven research based on ‘out-of-the-box’ thinking” (ibid). The Leiden Manifesto emphasizes that time is crucial for ensuring research quality and highlights the financial implications: “Accurate, high-quality data take time and money to collate and process. Budget for it” (Hicks et al., 2015).

We understand the research assessment reform movement to have two main goals: firstly, to change the way research and researchers are assessed to ensure fairness, inclusivity, and diversity; and secondly, by doing this, to strengthen the research ecosystem in ways that benefit not just individual researchers, but also the quality and integrity of the research produced. However, if this is the goal, the issue of temporality in research must be addressed much more profoundly by the movement. As our analysis will show, promoting temporal autonomy (Vostal, 2016, p.196) and prioritizing process time (Ylijoki, 2013) are key to ensuring high-quality research and sound research integrity practices. We argue that if issues related to temporality are not adequately addressed by the reform movement, changes will only be superficial, failing to bring about real improvements in research quality and integrity.

The theoretical framework of our paper draws on recent studies of the role of temporality in late modern society and academia (e.g. Rosa 2010, 2013; Ylijoki and Mäntylä 2003; Ylijoki 2015; Vostal 2015a, b; Vostal et al., 2019; Müller 2014; Felt 2017). Several of these studies have already contributed to a general understanding of the role of temporality within academia. However, we still know little about the relationship between temporality and research integrity/quality. This is the motivation behind this study in which we analyse material from thirty-six focus group interviews on research integrity, involving 172 researchers of diverse national, institutional and disciplinary backgrounds (see Methods section for more details). We have analysed the interviews with a particular focus on researchers’ experiences of the relationship between time and good research practices.

The article unfolds as follows. First, we present our theoretical framework for studying temporality in research: “Social acceleration and high-speed academia”. Second, the paper gives a thorough introduction to current discussions on time-use in academia and reviews the most influential theoretical contributions on temporality. Third, we present the methods used and our analytical strategy. Fourth, we present our findings, focussing on researchers’ experiences of temporality in research, including consequences of not having enough time. Fifth, we discuss our findings in relation to the goals of the research assessment reform movement, before we summarise our findings and analysis in a conclusion.

## Social Acceleration and High-Speed Academia

One of the most influential contemporary theories of modernity is the German sociologist Hartmut Rosa’s theory of social acceleration (2010, 2013). According to Rosa, modern societies are characterised by an historically unprecedented increase in the speed of technological and social change that also accelerate the ‘pace of life’. Among the most important consequences of technological acceleration are the contraction of space, i.e., that physical distances lose their meaning because of developments in transportation and communication. Social relations likewise change at an increasing speed, and the experience of progress is substituted by a feeling of random, episodic, and hectic change without direction (2010 pp. 41–47). Rosa describes acceleration as a form of totalitarian regime and says that there is “… hardly any arena in social life which is not affected or transformed by the dictates of speed.” (Rosa 2010, p. 62).

New technological developments create possibilities and potentials for faster and more efficient communication, transportation, and production. However, these possibilities also quickly become embedded in social practices, institutions, and cultural norms. This integration shapes future expectations and demands for even faster and more efficient outcomes. In academia, the rapid advancements in digital and information technology are currently transforming both teaching and research and changing the pace of academic work life. In parallel, new organisational, managerial and administrative processes are restructuring academic knowledge production. This includes a rapid implementation of new socio-economic arrangements, such as audit mechanisms and micro-surveillance components, designed to increase the efficiency of specific tasks, actions, and executions related to academic employment and positioning. This development has been conceptualised by Ylijoki (2013) as ‘high-speed university’, while Vostal (2016) refers to ‘accelerating academia’ and a ‘changing structure of academic time’.

Today, changes and regulations of academic work also seem to be occurring more frequently than before, and they are often aimed at speeding up processes connected to academic labour (see Ylijoki 2013; Vostal 2015a). Accelerating actions becomes a temporal coping strategy in contemporary academia. New generative AI technology is the latest development in this regard, employed across the research process from idea generation, research design, data generation and analysis to publications and disseminations (Andersen et al., 2025; Sørensen et al., 2024). Because of the opportunities yielded by new information technology, instant access to resources has become the new standard. This is also the case for human resources. As academics, we are likewise expected to always be flexible and available. New technology like smartphones make immediate responses and flexible availability a new social obligation, also in academia.

## A Theoretical Framework for Understanding the Effects of Acceleration in Academia

Despite its evident merits as a compelling framework for understanding the human and social condition in modern societies, both generally and within academia, Rosa’s theory of acceleration has been partially criticized for being too general to capture the specific complexities of time issues related to scientific knowledge production. Felt (2017), for example, on the one side found strong support for Rosa’s general acceleration hypothesis within academia, but at the same time also emphasised that a theory of acceleration within academia must be able to incorporate examples of “micro-resistance and of successful efforts of local re-timing” (p. 141). Vostal et al. (2019) develop what they call temporal complexity in scientific knowledge (re)production. Without arguing against Rosa, they write that “… science is characterized by layers and levels of diverse temporalities, rather than by one type of temporality: either problematic and much criticized ‘fast academia’ fed by neoliberal reforms/ideology or, often relatedly, ready and normative accounts demanding ‘slowdown’ of science/academia.” (p. 793). To them, “Manoeuvring, (un)bending, (dis)connecting, responding and reacting to, (re)routing and (re)orienting both expected and unexpected tempos of experimental, cognitive and institutional nature constitute a fundamental feature in the process of scientific knowledge production.” (p. 799). In an earlier article, Vostal (2015a) investigated the tension between different forms of time requirements and time-desires – fast and slow – in academia and especially analysed the feeling of guilt that academics can experience as a result of not being able to decide the pace of their own work and because of the prioritisations they are forced to make.

The differences between different time requirements are also at the centre of Ylijoki and Mäntylä’s (2003) interview study. They interviewed 52 Finnish researchers, analysed their experience of time in academia, and constructed four analytical categories/experiences of time: (1) ‘Scheduled time’, which is the externally imposed and controlled timetables (i.e., meetings, deadlines, supervision, lecturing hours, admin etc.). According to Ylijoki and Mäntylä an important feature of this form of time is its accelerating pace. There is a feeling of having to do more and more of these externally imposed tasks. (2) In stark contrast to scheduled time is ‘timeless time’, where time is internally motivated and clock time loses its importance. This time relates to researchers’ enthusiasm and fascination with their work and sometimes also to a feeling of being in ‘flow’. For most of the interviewees ‘timeless time’ is seen as an ideal, something they might have experienced when working with their PhD thesis, could still occasionally encounter, or aspire to experience in the future, when they have fulfilled their current obligations. (3) Ylijoki and Mäntylä also discuss a third form of time, called ‘contracted time’. This form of time is connected to the experience of time in relation to fixed-term research and work contracts. It includes a strong focus on the time that is left of one’s current contract, and the opportunities for getting a new contract. This form of employment gives the researchers little time to rest or make long-term plans. There is a strong focus on achievable results within the contract period and to funders and employers. According to Ylijoki and Mäntylä, the growing number of fixed-termed researchers means that the “dominating time perspective in an academic unit are temporary, episodic and contracted”. (4) Finally, Ylijoki and Mäntylä also identify a fourth form of time called ‘personal time’. This form of time is closely connected to the finitude of human existence, to life and death. It has to do with the time researchers spent on themselves, their families, and friends. With a reference to Arlie Hochschild, Ylijoki and Mäntylä note that researchers’ often end up having to steal time from their families and themselves to fulfil their obligations (scheduled time, contracted time) and to be able to experience ‘timeless time’.

In Ylijoki (2015), the meaning of contracted time - here referred to as ‘project time’ - is explored further. Ylijoki makes a distinction between ‘project time’ and ‘process time’. According to Ylijoki, we have experienced a projectification of the whole of society, and not least of academia. The project format has become a standard way of organising research and Ylijoki describes it (with a reference to Barbara Adam) as dominated by the four Cs: commodification, control, colonization, and compression of time. Ylijoki analyses the tension between the clock controlled ‘project time’, which she also describes as linear, cumulative, and progressive, and ‘process time’, which is blurred, obscure, and unlimited. She summarises the difference: “… project time represents the temporality inherent in the project format, which has become the dominant method for organizing academic research (and several other activities) at the present-day university. Process time, embedded in the internal logic of research activity, stands in many respects in opposition to project time.” (Ylijoki, 2015, p. 97).

As shown in the Findings section, lack of time and experienced time pressure are important concepts in our study, applied by the interviewees to explain why research integrity can come under pressure. Time pressure can be understood in relation to Ylijoki’s concepts of project time and process time. If the time allocated to a particular piece of research (project time) is less than what is needed to carry out the research (process time), then a researcher will experience time pressure. However, time pressure can also be related to what Müller (2014) calls ‘anticipatory acceleration’ in her study of early career researchers within the life sciences. She defines ‘anticipatory acceleration’ as a “[…] type of acceleration that aims at increasing units of output per units of time that is not developed in response to a certain event or specific time horizon, but rather constitutes a generalized response to a state of pervasive competition […]” (Müller, 2014). The feeling of time pressure is here connected to the general competition within academia and can be understood as a side-effect of career concerns, i.e., of the anticipation of the research output needed for having a career in academia. As Müller puts it: “Under the conditions of anticipatory acceleration everyday time is always scarce and desired to be devoted to those activities that can increase the output per time ratio.” (Müller, 2014). Our findings show that this is a widely shared experience across academia, and that it in some cases jeopardizes research integrity.

Although the need for a general slowdown of research activities and the restoration of ‘unhasty time’ (Pels, 2003) seems desirable as a cure for existing metrics, quantification of research, and managerial pathologies – such as those targeted by the research assessment reform movement (Rushforth & Hammarfelt, 2024) – many researchers, especially senior ones, acknowledge that speed and acceleration can have positive aspects. The relationship between academics and acceleration is quite complex. While many academics resent the increased speed, others strategically manage it and even find it thrilling and productive (Vostal 2015a, b). Therefore, instead of arguing in favour of slow science and slow academia in general, we will attempt to further develop the notion of temporal autonomy (Vostal, 2016, p.196) in this paper. This concept recognises the right to individually and collectively set temporal horizons for academic research production that better accommodate the needed ‘process time’ than what current academia allows. We will further discuss this concept and its potential for the research assessment reform movement in the Discussion. Here, we will argue that searching for ways to safeguard – or reestablish – temporal autonomy could be a productive focus for the reform movement.

## Methods and Analytical Approach

Although indicators suggest an increase in the pace of academic life – where more activities must be managed per unit of time than previously – this paper adopts what Rosa describes as a subjective approach to the study of time and research integrity (2010, p. 21). We focus on researchers’ experiences and perceptions of time pressure, acceleration, and research integrity in relation to the current research assessment system. This approach aims to provide insights into the way in which researchers think about their own and others’ research practices, including their perceptions of the current research assessment system and its effect on contemporary knowledge production.

The analysis presented in this paper draws on a unique set of data from two focus group studies that explored research cultures and practices in relation to conditions, challenges, and requirements of research integrity. The analysis is based on material from 36 focus group interviews conducted across seven European countries, involving a total of 172 researchers.

The first focus group study consisted of 22 focus groups. These focus group interviews were conducted as part of the research project Practices, Perceptions, and Patterns of Research Integrity (PRINT, 2017–2019). The main objective of this study was to survey and explore the state and conditions of research practices, knowledge production, and research integrity, with a particular focus on researchers’ perceptions of and engagement with questionable research practices (QRPs) across eight Danish universities and all major research fields. The research fields included the humanities, medical sciences, technical sciences, social sciences and natural sciences. An overview of socio-demographic information is provided in Table SI1, along with methodological details on the research design, sampling, and recruitment strategy (see Supplementary Information).

The second focus group study was part of a European project titled Standard Operating Procedures for Research Integrity (SOPs4RI, 2019–2022). Its aim was to examine transformational research integrity processes within European research and funding organizations. The study conducted 30 focus group interviews across eight European countries, exploring how various research areas perceived and ranked a range of research integrity topics – such as research environments, collaborative research, and breaches of research integrity – relevant to both research-performing and funding organizations. Fourteen of the focus groups consisted solely of researchers, while the remaining 16 included both researchers and other relevant stakeholders. In the present analysis, we draw exclusively on material from the 14 focus group interviews with researchers. As in the first focus group study (PRINT), all major research areas were represented, including the humanities, social sciences, natural sciences (including technical sciences), and medical sciences (including biomedicine). Table SI2 in the Supplementary Information provides an overview of the 14 focus groups, including details on research area, stakeholder representation, and demographic characteristics (see Supplementary Information for a detailed account of the study’s methodological approach).

The analysis in this article focuses on researchers’ perceptions of pace and research integrity in relation to their lived experiences in academia and with current research assessment systems. Our work on the two above-mentioned studies revealed the importance of also understanding researchers’ experiences of temporality as a significant factor affecting research quality and integrity. While it was not possible to make a systematic analysis of the temporal dimensions in (Ravn & Sørensen, 2021 and Sørensen et al., 2021), the similar nature of the two studies (cf. description of these studies in Supplementary Information) allowed us to combine the datasets to address the research question: How do researchers across fields and disciplines experience and perceive the relationship between temporality, acceleration, and research assessment systems in academia, and what are the implications for contemporary knowledge production?

Researchers’ accounts across all focus group interviews include narratives, experiences, and examples of navigating contemporary academia across countries, institutions, research fields, and disciplines. Individually and collectively, these accounts highlight cross-cutting characteristics of the current research assessment system in relation to time (cf. Introduction) and its perceived positive and negative effects on contemporary knowledge production. The article focuses on the analysis of the consistent patterns identified across narratives of perceived acceleration of academic life.

Authors MPS and TR led the design, execution, analysis, and reporting of the original studies. The interview transcripts from the 36 focus groups were combined into a new dataset. Twenty-two of the transcripts were in Danish, and the remaining transcripts in English. All transcripts were coded in their original languages to remain as close as possible to the verbatim versions. The similar research designs of the two studies made it possible to integrate the two datasets. However, as one study was conducted within a Danish context, the combined dataset exhibits a slight overrepresentation of Danish researchers (cf. Limitations). All transcripts were re-coded in NVivo by author ML, guided by the main research question and operationalized through a set of sub-questions and a theoretically informed coding strategy (cf. ‘Theoretical Frame’). This strategy focused on a thematic, cross-case comparison of the focus group data. The coding strategy was informed by theories of acceleration in academia. We operationalised key theoretical concepts – such as ‘acceleration’, ‘competing temporalities’ and ‘projectification’ – by coding empirical practices, experiences and narratives of time using language close to that of the interviewees, for example: ‘acceleration of publication pressure’, ‘time constraints and impact on research quality’ and ‘time pressure and unrealistic expectations’. In addition, the coding strategy applied an exploratory and inductive approach, allowing for the emergence of cross-cutting themes relevant to our research question. The participants were pseudonymised, and quotations include only general information, such as type of representation, position, and gender, without disclosing any identifying details.

## Findings Section: Time pressure and Potentially Declining Quality

In this section, we first examine the general time pressure that researchers experience in their daily work. We then focus on the impact of this time pressure on: (a) the quality of the research produced, and (b) the quality assurance system within research. Subsequently, we explore additional research integrity problems – and a few advantages – observed by the interviewees in relation to time pressure and accelerated research processes (for other positive aspects of the acceleration of academic life, see Vostal 2015b). Finally, the section addresses the experienced reasons for accelerated research processes.

### Experienced Time Pressure

Across the focus groups, interviewees expressed a feeling of working under intensifying time pressure in contemporary academia, similar to observations made by Vostal (2015a, b) and Ylijoki (2013). Many interviewees felt they needed to perform more tasks today than previously, and that the allocated time per task was far too short. They reflected on experiences of time shortage and its impact on research from multiple angles and dimensions. Regarding their research, interviewees expressed having too little time to read, collect new data, conduct in-depth analysis, comply with relevant regulations, observe due diligence, and supervise and mentor. In the words of Ylijoki (2013), they missed the necessary ‘process time’ for their academic work. Many researchers found it difficult to simply find time to do research.

Below, we will explore different forms of time pressure experienced by researchers in relation to various phases of the research process. However, before examining these phases, it should be noted that interviewees generally experienced a considerable mismatch between expectations and the time available to carry out set tasks. The clash between research and teaching time, as well as research and application time, especially stood out in the interviews. Writing applications and teaching often steal time away from research, as illustrated by this quote from a female Danish assistant professor in the social sciences (translated by the authors):

Interviewee: “Yeah, so we have two and a half hours of preparation time for a one-hour lecture, right? And I really think so - I spend an enormous amount of research time preparing lessons, and I don’t think I’m the only one who does that.”
Interviewer: “So when do you do research?”
Interviewee: “Well, Sunday evenings (mild laughter).”

Whenever there is a conflict of time between tasks, research seems to have to give way to the other responsibilities. As an interviewee on a temporary contract explains: “I’ve been teaching actually for a couple of years now and I’ve had no time for publishing, so I am actually now waiting for my third contract to run out so that I’ll have this obligatory time off to actually do the writing and then hope to get a position somewhere.” (Lecturer, humanities, the Netherlands). However, even though many of the interviewees experienced having very limited time available to do research and write papers, they still felt compelled to publish “a lot” to remain in academia: “[…] so that’s also what you’ll be measured on next time you have to have a position: How many articles has she published?” (Postdoc, natural science, female, Denmark, translated by authors).

### Time Pressure and the Risk of Declining Quality

Across disciplinary fields and countries, focus group participants expressed a feeling of being under double pressure. On the one hand, they feel they must carry out more tasks today than before, with less time for each task compared to earlier stages of their academic careers. They especially experience a lack of time to conduct research and to maintain high standards. On the other hand, they simultaneously experience high publication pressure – a feeling of having to publish more than previously to succeed in academia (to secure grants, permanent positions, promotions, or simply to continue their careers and research trajectories).

The experienced pressure leads to a general feeling of lacking time to adhere to norms of good research practices within the researchers’ different fields, which is perceived to be potentially harmful to the quality of the research produced. The pressure to publish is generally understood to manifest in prioritizing quantity over quality, as also emphasized by the research assessment reform movement (cf. the Introduction). Interviewees observed detrimental effects on research quality across the entire research process. To shed more light on this, we will use an analytical model for the research process encompassing five phases (cf. Ravn & Sørensen, 2021): the idea generation phase, research design phase, data collection phase, data analysis phase, and research publication and reporting phase.

In *the idea generation phase*, interviewees stated that they lack time to come up with new ideas, and that it is safer for them to reuse old ideas. They also feel they have too little time to read the existing literature before starting a new project: “[…] when you are under a lot of pressure, as we all are due to a large workload, then there is a tendency for people not to have enough time to read the literature within the field properly.” (Professor, humanities, female, Denmark, translation by authors). This is perceived to lead to studies that are too similar and make minimal contributions, ultimately failing to advance the field.

In *the research design phase*, interviewees point out that time pressure leads to fewer ‘high risk, high gain’ studies: “The worst problem with all this publishing pressure is all the stuff that doesn’t get done. We don’t choose a research area with high risk, because we know too well that there is not much chance of the t-statistics becoming significant.” (Associate professor, social science, male, Denmark, translation by authors). The experienced publication pressure creates a feeling of not having time to pursue riskier research areas, i.e., areas with more uncertainty where one risks ending up with negative results that are difficult to publish. Furthermore, interviewees – such as this Danish humanities scholar (professor, male) – express a feeling of not having time to use more advanced or ambitious methods (translated by the authors):

“Interviewee: I love doing these long historical analyses, but it is extremely time-consuming. You have to sit and rummage around in various archives and so on. There is no time for that. So what it ends up with often is that I look at what others have done of historical analyses and then I start to cannibalize a bit on this. And sometimes I work from there and find some data myself, right? But sometimes I end up doing a reinterpretation of the historical analyses, and see them in a different light, right? Sometimes I add a few additional sources, instead of starting from scratch myself. I definitely don’t have time for that.
Interviewer: It makes for poorer research?
Interviewee: I think so. In any case, it does not produce the research with originality that could have been done if I had had the time to do it.”

Like this scholar, many interviewees express a feeling of not having enough time to collect the most relevant data during *the data collection-phase*. Due to the perceived pressure to publish, stemming from institutions’ bibliometric point systems and career-related competition, data collection risks becoming an activity where researchers focus more on time constraints than on collecting the most optimal data for a given research project:

“There, I mostly just think that I have to get those points [for the bibliometric assessment system used in this institution], and how can I do that most easily - meaning, how can I get as much as possible as fast as possible, what kind of data can I most easily access? Instead of thinking I have to do field work for three months in [name of place] again, I don’t have time for that at all. […] Then you think creatively that way, and it is not necessarily questionable research, because it can be both real and valid. But you think in a different way, because there is this [name of bibliometric assessment system …]. For me, it affects what I study.” (Assistant professor, humanities, female, Denmark, translated by authors).

Career considerations – competition for grants, positions, and promotions – can override concerns over the research quality and determine the perception of how much time one can afford to spend on data collection. In a focus group within clinical-translational medicine, an interviewee described his experience of a sped-up contemporary research culture by recounting a younger colleague’s reaction to a story about a clinician who spent four years collecting data for a study. The young colleague’s reaction to the story is also a perfect example of what Müller (2014) calls ‘anticipatory acceleration.’ According to the interviewee, the young colleague said:

“Shit, he [the clinician] must be stupid. Spending so much time collecting that data to be able to write those two articles. That’s completely crazy. Over here, we can do this and that and that, and then we have 10 articles. And if you haven’t done that, then you’re already behind from the start […]” (Associate professor, medical science, male, Denmark, translated by authors).

In *the data analysis-phase*, interviewees similarly pointed to multiple examples of suboptimal research processes due to a lack of resources and a feeling of time pressure and competition: “[…] the vast majority of research groups do not have the resources to really check things through and to spend too much time on it, and some of the groups that do really well do so precisely because they don’t […]” (Associate professor, medical science, male, Denmark, translated by authors).

In individual projects, career considerations and the ‘projectification of research’ (Ylijoki, 2015) are seen as the primary reasons for using suboptimal processes. Researchers can feel they must think more about finishing on time than using the necessary time to conduct the research thoroughly, including validating results:

“But what I have experienced myself and around me is that there is sort of a time pressure especially when your PhD contract is almost finished. So, maybe at some point, you don’t do this extra analysis, you don’t extra check these outputs. And so, I feel like maybe it’s the risk in my case or around me is more like sloppiness a little bit. Because you just want to be very efficient with your time.” (Postdoc, medical science, female, the Netherlands).

Using Ylijoki’s (2015) terminology, the interviewee points to a mismatch between project time (finishing on time) and process time (doing the extra checks and analyses). Like most interviewees in our study, they clearly feel that project time trumps process time whenever there is a conflict between them.

Finally, if we look at *the publication and reporting phase*, interviewees here feel they have too little coherent time for writing. The lack of time leads to a rushed process, resulting in a lack of finesse in the form – such as writing style – to more profound impact on the content of the publication. This issue is also present in collaborative projects, where it can be challenging to find the time to thoroughly discuss and integrate different parts of a publication before submission:

“It becomes more like that we just share the work, “you do this, you do that”, and then we put it together, and then of course we all read it through. And then it’s often the case that it takes off in three different directions, because we didn’t have time to sit down and thoroughly discuss the concepts and how to do it.” (Professor, humanities, female, Denmark, translated by authors).

### Time Pressure and the Risks Posed to the Quality Assurance System

As the previous subsection shows, there is a prevailing feeling among the interviewees that the quality of the research produced may be harmed by the time pressure they experience. However, time pressure not only diminishes the quality of the research produced, it also potentially harms academia’s quality assurance system, particularly the peer review process. One interviewee, who is also a journal editor, describes this development in the following way:

“I’m an editor for a couple of journals, and to me it’s very difficult to find good reviewers, even to find reviewers is very difficult because people think that this is a waste of time, or some people think that this is a waste of time, and they prefer to work on their own studies, ignoring that the peer review process is the basis of the system, is the way science works. And also in the way the reviewers look at the papers, sometimes there is clear conflict of interest that editors not always deal with. Uhm and the quality of reviews is not always the quality that you should expect.” (Senior researcher, natural science, male, Spain).

Some senior academics deal with the time pressure related to peer review by accepting review tasks but passing them on to their PhD students or postdocs. In some cases, senior researchers take the time to discuss the strengths and weaknesses of a paper with their younger colleagues; in other cases, they let the PhD students handle the reviews without any guidance. Generally, time pressure can also lead to insufficient supervision of PhD students. Due to competition and prestige, the number of PhD students that some senior academics supervise is increasing, potentially negatively impacting the quality of supervision. Interviewees describe how the lack of time for proper supervision leads to PhD students submitting substandard papers to journals and conferences.

The lack of time to thoroughly review PhD students’ research and conduct in-depth journal peer reviews also affects the hiring and appointment processes within academia. When applicants submit papers as part of their application, assessment committee members often do not have the time to read the papers. Instead, they rely on metrics, despite knowing that this is a suboptimal way of evaluating researchers’ work:

“And if I’m in a panel of reviewing some applications or something like that, I do get credited a little bit of money, but I have so little time to do it, I mean, it’s just not. And that’s the reason why we end up focusing on these index numbers, which is bullshit of course.” (Associate professor, natural science, male, Denmark).

Finally, when researchers notice potential integrity problems in the work of their peers, they do not always react due to time constraints. When they do address these issues, it is often experienced as very time-consuming: “Everybody forgets how time consuming it is to prove misconduct. It takes months of work.” (Senior researcher, medical science, male, Greece).

### Further Experienced Impact on Research Integrity

When listening to the interviewees in our study, there is no doubt that accelerated research processes and time pressure are generally perceived to harm research integrity. Researchers across various disciplines find it challenging to adhere to norms of good research practices due to these pressures. Although, as we shall see below, the current system also leads to changes in practice that can be beneficial to research integrity, the main narrative emerging from our study is that time pressure and accelerated research processes are detrimental to research integrity. Some interviewees directly associate these processes with the use of questionable research practices (QRPs), or suggest that QRPs become more attractive in a system focused on speed and high publication pressure:

“I’m afraid we work in a very high-tension field, and everybody is forced to publish as quickly as possible to become eligible for the grants, and yeah I’m afraid this is where integrity becomes damaged […] Yeah, p-hacking, and I guess really people would be tempted to go into the grey area. Maybe not outright fraud, but: ‘Can I remove an outlier still?, Can I test this in another way?, or coming up with hypotheses after the data has been gathered” (Assistant professor, social science, the Netherlands).

Time pressure might tempt some researchers to manipulate their analyses to show stronger positive effects, ensuring the publication is accepted and avoiding the loss of time that would result from a failure to publish:

“(…) you cannot publish anything which doesn’t show effect, or something. So, in order to get publications which are necessary, and because they’re an indicator of your performance, there is a really strong incentive, in my opinion, for scientific misconduct, for faking data …” (Senior researcher, social sciences, Germany).

The current merit system is also understood to encourage researchers to publish small and insignificant papers: “[…] we are flooded with publications that don’t matter to anyone.” (Associate professor, natural science, male, Denmark). The pressure to publish is experienced to lead to an overproduction of publications with very limited contribution or novelty: “One basically produces ten times too many or 50 times too many articles. […] They are just too close to each other. There is too little new.” (Associate professor, natural science, male, Denmark, translation by authors).

Unfair assignments of authorships and authorship conflicts also seem to flourish in this system. Strong focus on quantity and researchers’ need to boost their publication lists turn authorship discussions into a form of gift economy:

“[…] there is a clear tendency of people saying implicitly that ‘if you put me on your paper, I’ll put you on my paper’, and then you see these papers with 10 or 15 authors, and essentially there is one who has done all the work, and the rest is just on for the ride. The problem is […] whoever has the most publications win and we need to break that, otherwise things are gonna move in a really bad direction.” (Associate professor, natural science, male, Denmark).

In fields with multiple authors, such as the medical sciences, authorships can be used as a form of currency that can repay old favors or make collaborations run more smoothly: “[…] we depend on the [name of] departments […] and sometimes that means that you add an author to a paper where the contribution is a bit, uhm, less than 5% […]” (Associate Professor, medical science, female, Denmark).

However, alongside the main narrative that time pressure and accelerated knowledge production harm research integrity, a secondary theme emerged in our interviews. Some participants from both humanities and natural sciences noted that this development, despite its drawbacks, also brought certain benefits. This secondary theme emerged during discussions on increasing publication rates, the peer review process, and the pressure to publish at different career stages. For example, a natural science researcher pointed out that the increased focus on publications had given PhD students the opportunity to learn how to write papers and practice their writing skills. In the humanities, several scholars noted that the shift from writing articles and books in national languages to focusing more on international journal articles – stimulated by new bibliometric-focused research assessment systems – had led to more widespread use of peer review within the humanities. This, in turn, was perceived to have improved the overall quality of publications within various fields in the humanities:

“I would say that, on the one hand, there is a tendency to proceed quickly and produce as much as possible, […] But it is also within the past ten years that peer review has become more common with us, compared to what it used to be. It has also led to an increase in quality, whereas some things that would have slipped through in the past, now these are weeded out” (Associate professor, humanities, male, Denmark).

### Experienced Reasons for the Increased Time Pressure

But what is causing the pervasive feeling of time pressure in relation to research tasks? As already mentioned, many interviewees perceive teaching to take up more time than allocated, thereby eating into research time. Additionally, as universities become more dependent on external funding, researchers now have to spend more time applying for grants than before: “[…] less time is actually spent on the actual research and more on writing applications and the competition we all know is getting harder and harder.” (Associate professor, humanities, female, Denmark). The combination of increasing demands for external funding and decreasing success rates puts extra pressure on researchers’ time. However, winning a grant does not necessarily alleviate pressure or provide more time for individual steps in the research process. On the contrary, winning a grant might lead to even more time pressure: “In order to get proposals accepted, you have to promise to do more than you actually think you can achieve.” (Postdoc, technical science, male, Denmark, translation by authors). Early career researchers, in particular, seem to be under significant pressure to secure the right grants to advance in the research system: “[…] when you finish your PhD, it’s ‘make it or break it’, […] then you have two years to create the results or the CV that will get you the job, because there are fewer and fewer grants to apply for when you get a little further [into the postdoc period].” (Associate professor, natural science, male, Denmark, translated by authors). If they are successful in securing a grant, the pressure then comes from delivering the results they promised in the application: “[…] if you are a young group leader - this does not only apply to Denmark, it applies to the whole world - then you are typically hired on some interesting hypothesis, or some other interesting concept, and then it has to work, that is, if it doesn’t work […] then it is almost over for you.” (Associate professor, medical science, male, Denmark, translation by authors).

When listening to the interviewees, it becomes clear that time pressure is closely linked to competition. Competition for grants, positions, and promotions leads to ‘anticipatory acceleration’ (Müller, 2014) and an experience of time pressure. As one of the interviewees explains: “as soon as you become a PhD - it’s not nice, but - you’re competing with your colleagues, you’re competing with the other PhDs because there’s only going to be so many assistant professor positions, and after assistant professor there are only so many in the tracks, so I think the pressure with us is very real.” (Assistant professor, social science, the Netherlands). The career system is further tied to a general projectification (Ylijoko, 2015) of academic work, including research:

“To me, that’s where the pressure lies. It’s in how quickly you have to get things out. Also, because there… Now again, I think it is very typical for research careers that there are many projects, both project assignments and that things are generally very project-driven, where there is a clear deadline for when things must be finished. That might put extra pressure on top.” (Associate professor, humanities, male, Denmark, translation by authors).

The project format has thus become a standard modus operandi in academia (Ylijoki, 2015; Felt, 2017). At the point of conception, studies must accommodate the project format, adhering to fixed timelines, predictability of findings, the feasibility of positive results, and inflexibility of research design. Working in project-related temporary positions also means always having to consider your next position. In this way, one’s current research practice becomes – at least partly – shaped by the perception of what will secure one’s next position.

## Discussion

An important finding in our study – one that also supports the research assessment reform movement’s call for a change in the way we assess research and researchers (cf. ‘Introduction’) – is the detrimental effect that current merit and reward systems are perceived to have on scientific progress and the quality of our collective knowledge production. Like Edwards and Roy (2017), we find that problems with robustness and lack of reproducibility of scientific results (see e.g., Ioannidis, 2005; Baker, 2016; Resnik & Shamoo, 2017) are associated with current reward and merit systems in academia. As our interviewees pointed out, quality and integrity do not carry the same weight as quantity and speed in current systems.

This suggests tensions within current reward and merit systems, as also discussed by Aubert Bonn and Pinxten (2021). Behaviour that benefits the quality of the research produced – that makes research more robust and trustworthy – does not always align with metrics that advance individual researchers’ careers. On the contrary, investing extra effort to ensure high research quality may actually hinder career advancement by reducing publication output and making researchers seem less productive by conventional metrics. This means that researchers may face practical dilemmas where investing additional time in making their results more robust – such as by collecting additional data or conducting extra validation tests – may be disadvantageous to them in a system that prioritizes publication quantity.

Despite such conflicting rationales within current merit and reward systems, the interviewed researchers still seemed to care deeply about the quality of the research they produced. They were aware of acceptable standards and often went to great length to find the necessary time to meet the increased publication demands they experienced, while at the same time making sure that their research adhered to acceptable quality standards. This often involved sacrificing leisure time or family time to ensure they had enough ‘process time’ for their research. However, the interviewees were also acutely aware that researchers who prioritise quality and integrity risk losing out to those who focus on quantity and speed in the competition for positions, promotions and grants.

Whether funding bodies and hiring committees actually place more weight on quantity and speed than on quality and integrity in their decision-making is debatable. However, irrespective of objective practices the mere perception among researchers that this is the case can affect researchers’ behaviour and significantly influence how research is produced. Studies have shown that only a small percentage of researchers engage in the most serious forms of scientific misconduct – fabrication, falsification and plagiarism (FFP) – but that questionable research practices (QRPs) are much more widespread (Steneck, 2006; Fanelli, 2009; John et al., 2012; Anderson et al., 2013; Bouter et al., 2016; Gopalakrishna et al., 2022; Schneider et al., 2023). Recent surveys also show a positive correlation between researchers’ perceptions of the pressure to publish and attract external funding and their self-reported use of QRPs (Schneider et al. 2023; Gopalakrishna et al., 2022).

Based on the findings in this paper, the appeal and frequent use of QRPs may be partly attributed to their potential to accelerate the research process and reduce the time required to transition from research ideas to publications. Different QRPs are employed to expedite research processes across various research fields (Ravn & Sørensen, 2021). In the Humanities, they include plagiarism-like behaviours such as adopting existing structural frameworks from other studies. Insufficient reading of existing literature and inadequate engagement with state-of-the-art research are other practices that can serve to expedite the path from conception to publication.

Medical and Natural Sciences reveal similar patterns, with examples including omitting necessary controls of reagents or animal models and failing to critically validate results. Technical Sciences show comparable practices in result validation. Another widespread QRP across these fields is ‘salami slicing’, i.e. fragmenting research into multiple publications to inflate publication records. Within the Social Sciences, QRPs accelerating the research process also include deliberately avoiding time-intensive methodologies.

However, it is crucial to recognize that the wish to speed up the research process is not the sole driver of QRPs. Researchers’ pursuit of recognition and prestige through the production of spectacular results also seem to motivate QRPs. Prevalent practices such as cherry-picking data sources, selective reporting of favourable findings, and p-hacking might be examples of this kind of motivation. Additionally, some QRPs arise from inadequate training, where researchers remain unaware of the detrimental consequences of their practices rather than consciously choosing shortcuts. A core example of this could be ‘Inadequate use of methods’ (Ravn & Sørensen, 2021).

For individual researchers and research groups, speeding up the research process using QRPs can be attractive and might even be seen as necessary in a highly competitive research system. However, while speed-oriented QRPs may help individual researchers achieve personal goals faster, they create a paradoxical effect on collective scientific progress. Research conducted through such accelerated practices often lacks originality and methodological robustness, contributing minimal value to our shared knowledge base. Consequently, what appears to save time at the individual level ultimately wastes collective resources and slows down scientific advancement. This might also create tension between personal academic success and meaningful contribution to knowledge production, suggesting that current incentive structures may inadvertently reward practices that can undermine the very goals they purport to serve (cf. also Edwards and Roy’s, 2017, discussion of ‘perverse incentives’ in a ‘hyper-competitive’ science system).

Returning to the research assessment reform movement discussed in the Introduction, our findings clearly support this movement’s call for developing new ways of assessing research, based on fairness, inclusivity, and a broader understanding of what constitutes good research. However, our findings also indicate that the movement needs to be mindful of issues of temporality as part of this reform work. To enhance the quality of the research produced, the research system must provide opportunities for a greater focus on ‘process time’ rather than just ‘project time’ (Ylijoki, 2013). Knowledge production processes are characterised by both predictable and unpredictable time requirements, making standardized timeframes for research projects undesirable. We therefore recommend experimenting more with ‘temporal autonomy’ (Vostal, 2016).

An important *first* element would be to strive for a better alignment of ‘project time’ with ‘process time’, including acknowledging that research tasks vary in time. There is no standard time consumption per project, per research task or per paper. Funders and research institutions must create systems and procedures that recognize and are able to handle such temporal diversity. This means developing new procedures and cultures for evaluation of research, where assessors look beyond mere numbers of publications and focus more on the quality of the research, including assessing how time-consuming the methods used are or how much time went into data collection.

Related to this, a *second* recommendation is to develop new funding schemes and research evaluation systems that take into consideration that knowledge production processes are rarely straightforward but characterised by differences in tempo and research rhythms. Funding schemes and assessment criteria must be flexible enough to accommodate such temporal changes. Funders, for example, could become more open to extending research projects that have run into unforeseen challenges but still have the potential to produce interesting new knowledge. In general, it would be beneficial to implement more flexibility in funding schemes as an alternative to currently prevalent fixed-term approaches. This recommendation could also take form in funding schemes that provide basic support for an initial, short research period, giving researchers time to develop ideas and carry out initial experiments, before additional funding decisions are made based on qualitative assessments of the research’s potential and content.

A *third* crucial aspect, related to the two previous suggestions, is to ensure researchers and research units have adequate time to balance research completion with responsible conduct – without compromising resource efficiency. Research proposal assessments should also systematically evaluate the relationship between proposed timelines and research quality. When evaluating research proposals, assessors should critically examine whether proposed timelines are realistic for the expected output and whether they allow sufficient time to uphold high research quality and integrity standards. Assessors should be particularly wary of attempts to oversell or overpromise results. Such attempts will only inflate general expectations of how much one is supposed to produce within a given timeframe, which again can jeopardize the quality of the research produced and the integrity of the research process. Similarly, post hoc assessments should focus more on how the funded time was utilised, especially recognising time invested in improving the robustness and quality of the research results.

### Limitations

The focus group interviews that form the basis of this paper’s analysis do not allow us to examine differences in perceptions of time-pressure and acceleration across institutional contexts, nationalities, seniority levels, or gender. Our focus groups were sampled to investigate disciplinary differences in the perception of research integrity (specifically between humanities, social sciences, natural sciences including technical science, and medical science including biomedicine), these disciplinary differences have been systematically explored elsewhere (Ravn & Sørensen, 2021; Sørensen et al., 2021).

In the present study, we used the same focus group interviews to examine shared perceptions of time and acceleration across disciplines, institutions, nationalities, seniority levels, and gender. This focus on shared patterns, while valuable for understanding broader trends, means that potentially significant variations remain unexplored. For example, there might be important differences between researchers working in different countries or institutions that reflect varying reward and merit systems – differences our study’s design cannot capture.

## Conclusion

In this paper, we have analysed experiences of time and temporality in academia and their perceived effects on research quality and integrity. Temporality is entangled with contemporary merit and reward systems – and fuelled by a generalised system of competition. We have therefore focused our analysis on the focus group participants’ experiences of these reward structures in academia, their temporal consequences, and the perceived implications for research conduct and research quality.

Our study of 36 focus group interviews across seven European countries in which 172 researchers took part revealed a remarkably consistent view of reward and merit systems and temporal regimes in contemporary academia. These views transcend differences in disciplines, country, institutions, gender, and seniority. The perceptions also align with the ‘research assessment reform movement’ (DORA, CoARA etc.). Interviewees described current systems as prioritising speed and quantity – emphasising rapid production of results, papers, and citations – partly at the expense of research quality and integrity and thereby potentially harming the robustness of the knowledge produced in contemporary academia.

While our findings support earlier studies (Aubert Bonn & Pinxten, 2021; Edwards & Roy, 2017), they also provide a deeper insight into researchers’ experiences of the connection between time and research integrity. Our study suggests that the research assessment reform movement needs to consider temporality in its efforts to achieve its dual objectives: reforming research assessment to promote fairness, inclusivity, and diversity while strengthening the research ecosystem to benefit both individual researchers and knowledge production quality. We recommend that funders shift their focus from ‘project time’ to ‘process time’ and that the reform movement emphasise ‘temporal autonomy’ to ensure researchers and research units have adequate time to carry out the research while maintaining integrity.

Our study also offers a new perspective on questionable research practices (QRPs). We argue that their widespread use stems not only from current reward and merit systems but also from the pervasive feeling of acceleration in academia, what Müller (2014) calls ‘anticipatory acceleration’. The combination of quantitative assessment metrics and perceived time pressure can make QRPs that accelerate research appealing to some researchers. Reducing QRP prevalence therefore requires both changing researchers’ perceptions of what matters in academia and moving toward more qualitative research assessment systems. Our findings thus support the reform movement’s efforts to balance quantity and speed with quality and integrity in research assessment.

## Electronic Supplementary Material

Below is the link to the electronic supplementary material.

Supplementary Material 1

## Data Availability

The data (14 focus group interview transcripts) from the SOPs4RI study are publicly available at https://osf.io/e9u8t/files/osfstorage (cf. FG1, FG4-6, FG10-11, FG15-17, FG20-21, FG23, FG25, FG30). The data (22 focus group interview transcripts) for the PRINT study are not publicly available since no consent was given for this by the participants.
