# Supplementary material for: Time, Quality, and Integrity: Temporal Autonomy as a Missing Link in Research Assessment Reform
Source: Sci Eng Ethics. 2026 Feb 27;32(2):16. doi: 10.1007/s11948-026-00588-x (PMC13009029; doi:10.1007/s11948-026-00588-x)
Supplement: Supplementary file 1 — Supplementary Material 1 [file 11948_2026_588_MOESM1_ESM.docx]

**Supplementary Information**

The objective of this study is to examine how researchers across fields and disciplines experience and interpret the interplay between temporality, acceleration, and research assessment systems in academia, as well as their implications for contemporary knowledge production. The analysis is based on a dataset drawn from two focus group studies exploring research cultures and practices, with particular attention to the conditions, challenges, and requirements related to research integrity. The following sections provide an overview of the socio-demographic characteristics of the participants in each study, along with details on the specific research design, sampling methods, and recruitment strategies. Authors MPS and TR led the design, execution, analysis, and reporting of the original studies.

**Focus group study 1. Practices, Perceptions, and Patterns of Research Integrity (PRINT, 2017–2019)**

The focus group interviews were conducted by authors MPS and TR as co-moderators in October, November, and December 2017 at eight universities in Denmark: University of Copenhagen, Aarhus University, Technical University of Denmark, Copenhagen Business School, Roskilde University, Aalborg University, University of Southern Denmark, and the IT University of Copenhagen. The study explored the following three research questions:

1. Which practices do researchers from the different main fields of research (humanities, social sciences, medical sciences, natural sciences, and technical sciences) identify as QRPs, and in what way do these QRP practices relate to different phases in the research process?
2. How do researchers from different research areas define and assess these QRPs?
3. To which extent do the QRPs relate to variation in research practices and ‘epistemic cultures’?

An overview of the 22 focus group interviews conducted, and the distribution of participants according to research area, gender and position can be found in Table SI1. Table SI1a provides an overview of the disciplines represented in the study.

**Table SI1. 1^st^ study. Groups and number of participants (distributed on gender and academic level)**

| Main areas of research | No. of groups | Participants | Female | Male | Prof. | Associate prof./senior researchers | Assistant prof./post  Docs |
| --- | --- | --- | --- | --- | --- | --- | --- |
| Humanities | 5 | 23 | 12 | 11 | 7 | 11 | 5 |
| Social sciences | 4 | 22 | 8 | 14 | 8 | 10 | 4 |
| Technical sciences | 4 | 19 | 5 | 14 | 3 | 8 | 8 |
| Medical sciences | 4 | 18 | 6 | 12 | 6 | 12 | 0 |
| Natural sciences | 4 | 20 | 5 | 15 | 5 | 11 | 4 |
| Interdisciplinary | 1 | 3 | 2 | 1 | 0 | 2 | 1 |
| Total | **22** | **105** | **38** | **67** | **29** | **54** | **22** |

Source: Ravn and Sørensen 2021.

**Table SI1a. Discipline Representation**

| Main area of research | Disciplines represented |
| --- | --- |
| Humanities | Linguistics, philosophy and history of ideas, history, aesthetics, communication |
| Medical science | Clinical medicine, forensic medicine, clinical epidemiology, clinical nursing, biomedicine |
| Technical science | Food, energy, environment, nanotechnology, photonics, engineering (biological, chemical, electrical, computer, mechanical, production) |
| Social science | Law, management, economics, political science, psychology |
| Natural science | Physics, chemistry, biology, geoscience, mathematics, pharmacy |

Source: Ravn and Sørensen 2021.

The focus groups were designed for homogeneity and focused on participants’ research practices - how science is conducted. Each group was designed to ideally include four to six participants with balanced gender representation and cover two to three sub-disciplines within each of the five main research areas. The goal was to ensure broad disciplinary representation across all major subfields. An information-oriented selection strategy guided participant recruitment, aiming for diversity across gender and academic rank (postdoc/assistant professor, associate professor, and professor). To encourage open discussion, senior/junior pairings from the same institute were avoided, and efforts were made to include participants with no prior research collaborations.

Participants were primarily identified through systematic reviews of university websites. Heads of department were notified of the study and informed that recruitment would occur via public university email addresses. This approach was supplemented by snowball sampling and outreach through the researchers’ own professional networks to identify participants not known by the interviewers. Recruitment proved challenging given the scale of the study with 22 focus groups in total. Out of 808 researchers invited, 105 participated, with group sizes ranging from three to six. For further details on the focus group methodology and moderator guide, see Ravn and Sørensen (2021).

**Focus group study 2. Standard Operating Procedures for Research Integrity (SOPs4RI, 2019–2022).**

The 14 focus group interviews from SOPs4RI that are used in the current study were carried out by six national SOPs4RI-partners during February, March and April 2020 in Denmark, Spain, the Netherlands, Germany, Belgium, Croatia, and Greece. The study explored the followed research questions:

1. Is there a need for different Standard Operating Procedures and guidelines in different disciplinary fields for the same research integrity topics/subtopics?
2. Which research integrity topics and subtopics are the most important ones for the different disciplines/main research fields (humanities, social science, natural science, and medical science)?
3. Do the different disciplines have any topics or subtopics to add to map of the research integrity landscape?

Table SI2 displays an overview of the distribution of participants in the 14 focus group interviews with researchers according to research area, discipline, country, position, and gender.

**Table SI2. 2^nd^ study. 14 focus groups on Research Performing Organisations. Participant characteristics.**

| Approach | Disciplines represented | Participants | Country | Seniority of researchers | Gender F/M (%) |
| --- | --- | --- | --- | --- | --- |
| *Humanities focus groups* | | | | | |
| Historical | History of ideas, Archaeology | 3 | DK | Assistant professor (1), Associate professor (1), Professor MSO (1) | F (33%), M (67%) |
| Language | Theoretical and applied linguistics, Sociolinguistics, Computer-mediated communication | 7 | NL | Postdoc (1), Assistant professor (2), Lecturer (2), Associate professor (1), Professor (1) | F (57%), M (43%) |
| Communication | Information science, Communication science | 6 | HR | Research assistant (1), Postdoc (1)  Assistant professor (3), Associate professor (1) | F (33%), M (67%) |
| *Social science focus groups* | | | | | |
| Qualitative | Sociology, Sociology and religion | 4 | ES | Postdoc (2), associate professor (1), senior researcher (1) | F (75%), M (25%) |
| Quantitative | Social psychology, Political science, Quantitative science studies, Education and child studies, Anthropology and developmental sociology | 6 | NL | PhD student (1), Postdoc (1), Assistant professor (3), Associate professor (1) | F (33%), M (67%) |
| Qualitative | Qualitative science studies, Higher education research | 5 | DE | PhD student (1), postdoc (1), junior researcher (1), Senior researcher (2) | F (60%), M (40%) |
| *Natural science (incl. technical science) focus groups* | | | | | |
| Experimental | Water management, Biodiversity | 2 | ES | Senior researcher (2) | F (50%), M (50%) |
| Theoretical | Theoretical physics, Mathematics, Chemistry, Computer science | 6 | DK | Postdoc (2), Associate professor (4) | F (33%), M (67%) |
| Experimental | Biology, Bioscience and engineering, Statistics | 3 | BE | Associate professor (1), Professor (2) | M (100%) |
| Experimental | Geoscience, Mathematics, Physics, Translational biomedicine, Biology | 6 | HR | PhD student (1), Postdoc (1), Senior researcher (1), Professor (3) | F (83%), M (17%) |
| *Medical science (incl. biomedicine) focus groups* | | | | | |
| Clinical | Clinical nursing, Oncology, Sexology | 3 | DK | Associate professor (3) | F (100%) |
| Clinical | Gastroenterology, Clinical epidemiology, Physiology, Vascular surgery, Clinical neuroscience | 6 | NL | Postdoc (1), Senior clinical scientist (1), Senior researcher (1), Professor (3) | F (67%), M (33%) |
| Basic | Forensic science, Histology and embryology, Anatomy, Neurobiology, Physiology | 7 | HR | Research assistant (1), Assistant professor (1), Associate professor (2), Professor (2), Professor emeritus (1) | F (43%), M (57%) |
| Basic | Medical law and ethics, Neurobiology, Biophysics | 3 | GR | Senior researcher (2), Professor (1) | F (33%), M (67%) |

Source: Sørensen et al. 2021

Of the 14 focus groups conducted across European countries, three were held in each the Netherlands, Denmark, and Croatia; two in Spain; and one each in Germany, Belgium, and Greece. Each group consisted of researchers from one of the four main research areas (humanities, social sciences, medical sciences, incl. biomedicine, and the natural sciences, incl. technical sciences), representing disciplines and core methodological and epistemic approaches specific to that area.

Eleven of the fourteen focus group interviews were conducted face-to-face, while three took place online due to cross-country COVID-19 lockdowns during the data collection period. All interviews were conducted in English. Participants were recruited based on the primary methodological approaches used in their research. In addition to this core sampling criteria, further selection criteria were applied to enhance representation, diversity, and heterogeneity:

- Each group should include both senior researchers in permanent positions (e.g., professors, associate professors, senior researchers) and junior researchers in non-permanent positions (e.g., postdocs, assistant professors, final-year PhD students).
- Gender balance should be maintained across all focus groups.
- Each focus group should represent two to three distinct sub-disciplines within the broader research area.
- The selected disciplines should provide broad representation of research conducted within the four main areas.
- Individuals who are professionally dependent on one another (e.g., a lab leader and an employee from the same lab) should not be included in the same focus group.
- All interviewees must be able to participate in the interview in English.

Researchers were recruited from universities and other research institutions. The focus group study employed a purposeful sampling strategy, based on the set of pre-defined criteria described above. In addition, a snowball/chain sampling method was used, where volunteers from existing and new networks were invited to act as gatekeepers, helping to identify and recruit relevant researchers and stakeholders within their organizations. This strategy was complemented by a randomized approach, in which participants were selected from institutional websites and subsequently contacted via email with an invitation letter. For further details on the focus group methodology, design and moderator guide, see Sørensen et al. 2020. All focus groups transcripts from this study have been made publicly available in anonymised versions to allow for secondary analyses of the data and to promote open data access (link to FG interviews: <https://osf.io/e9u8t/files/osfstorage>).
